# Genotype Imputation To Improve the Cost-Efficiency of Genomic Selection in Farmed Atlantic Salmon

**DOI:** 10.1534/g3.117.040717

**Published:** 2017-03-01

**Authors:** Hsin-Yuan Tsai, Oswald Matika, Stefan McKinnon Edwards, Roberto Antolín–Sánchez, Alastair Hamilton, Derrick R. Guy, Alan E. Tinch, Karim Gharbi, Michael J. Stear, John B. Taggart, James E. Bron, John M. Hickey, Ross D. Houston

**Affiliations:** * The Roslin Institute and Royal (Dick) School of Veterinary Studies, University of Edinburgh, Midlothian EH25 9RG, United Kingdom; † Hendrix Genetics Aquaculture BV/ NetherlandsVilla 'de Körver', Spoorstraat 695831 CK BoxmeerThe Netherlands; ‡ Edinburgh Genomics, Ashworth Laboratories, University of Edinburgh, EH9 3JT, United Kingdom; § Institute of Biodiversity, Animal Health & Comparative Medicine, University of Glasgow, G61 1QH, United Kingdom; ** Institute of Aquaculture, University of Stirling, FK9 4LA, United Kingdom

**Keywords:** aquaculture, disease resistance, Genomic Selection, imputation, GenPred, Shared Data Resources

## Abstract

Genomic selection uses genome-wide marker information to predict breeding values for traits of economic interest, and is more accurate than pedigree-based methods. The development of high density SNP arrays for Atlantic salmon has enabled genomic selection in selective breeding programs, alongside high-resolution association mapping of the genetic basis of complex traits. However, in sibling testing schemes typical of salmon breeding programs, trait records are available on many thousands of fish with close relationships to the selection candidates. Therefore, routine high density SNP genotyping may be prohibitively expensive. One means to reducing genotyping cost is the use of genotype imputation, where selected key animals (*e.g.*, breeding program parents) are genotyped at high density, and the majority of individuals (*e.g.*, performance tested fish and selection candidates) are genotyped at much lower density, followed by imputation to high density. The main objectives of the current study were to assess the feasibility and accuracy of genotype imputation in the context of a salmon breeding program. The specific aims were: (i) to measure the accuracy of genotype imputation using medium (25 K) and high (78 K) density mapped SNP panels, by masking varying proportions of the genotypes and assessing the correlation between the imputed genotypes and the true genotypes; and (ii) to assess the efficacy of imputed genotype data in genomic prediction of key performance traits (sea lice resistance and body weight). Imputation accuracies of up to 0.90 were observed using the simple two-generation pedigree dataset, and moderately high accuracy (0.83) was possible even with very low density SNP data (∼250 SNPs). The performance of genomic prediction using imputed genotype data was comparable to using true genotype data, and both were superior to pedigree-based prediction. These results demonstrate that the genotype imputation approach used in this study can provide a cost-effective method for generating robust genome-wide SNP data for genomic prediction in Atlantic salmon. Genotype imputation approaches are likely to form a critical component of cost-efficient genomic selection programs to improve economically important traits in aquaculture.

Modern genetic studies typically require high density genome-wide SNPs for mapping variants underlying complex traits, or predicting breeding values from genotype data. Genomic selection has transformed terrestrial and aquatic animal breeding programs, and relies on capturing accurate realized genetic relationships between animals, and linkage disequilibrium (LD) between SNP markers and causative mutations underlying economically important traits (Meuwissen *et al.* 2013). However, genotyping the large numbers of individuals required for accurate genomic predictions using high density SNP platforms is expensive, often prohibitively so. In turn, this can limit both the number of phenotyped individuals with high density genotype data in the training set used to derive the genomic prediction equation, and the number of selection candidates that can be evaluated using that equation (Meuwissen *et al.* 2001; Habier *et al.* 2009). The cost of genotyping is largely dependent on marker density, with low density panels being considerably cheaper than high density ones. Therefore, a targeted high and low density genotyping strategy in pedigreed animals, combined with genotype imputation, is an attractive option to improve the cost-efficiency of high resolution genomic studies, and application of genomic selection in aquaculture breeding programs.

Genotype imputation involves high density genotyping of certain key individuals, while the majority of individuals are screened only for a small subset of these markers (a lower density SNP panel). These genotype data are then used to impute the nongenotyped markers for the individuals genotyped at low density (Hickey *et al.* 2012a). Imputation approaches have been successfully and widely applied in breeding programs for several livestock and crop species (*e.g.*, Hayes *et al.* 2012; Hickey *et al.* 2012a; Pausch *et al.* 2013; Daetwyler *et al.* 2013; Moghaddar *et al.* 2015). The accuracy of imputation is affected by several factors, including population structure, the number of SNPs in the imputation panel, the level of relatedness between reference and test data, effective population size, the inherent accuracy of the method used for imputation, and the degree to which markers are correctly ordered along the genome map (*e.g.*, Hayes *et al.* 2012; Hickey *et al.* 2012a; Hozé *et al.* 2013; Uemoto *et al.* 2015). The methods applied for genotype imputation can broadly be split into two categories: (i) population approaches such as Beagle (Browning and Browning 2016), MaCH (Li *et al.* 2010) and IMPUTE2 (Howie *et al.* 2009), which utilize linkage disequilibrium (LD) between markers, and (ii) pedigree-based approaches such as PHASEBOOK (Druet and Georges 2010), findhap (VanRaden *et al.* 2011), and AlphaImpute (Hickey *et al.* 2012b), which harness genetic relationships (pedigree) in addition to LD. The latter approaches are suitable for data originating from typical livestock and aquaculture breeding programs, where large numbers of pedigreed individuals with genotype and phenotype data are routinely available.

While research into imputation methods and their application to breeding programs has been extensive for livestock and crop species, they have not yet been widely tested in aquaculture species (Kijas *et al.* 2016; Tsai *et al.* 2016a). In part, this is due to the previous lack of genomic resources (*e.g.*, SNP genotyping arrays and reference genome sequences) for many aquaculture species (Yáñez *et al.* 2014, 2015). In recent years, high density SNP arrays have been developed for several aquatic species, including salmonid species (Houston *et al.* 2014; Palti *et al.* 2015; Yáñez *et al.* 2016; Lien *et al.* 2016). These SNP arrays, alongside custom lower density SNP panels, have been successfully applied to enable genomic selection for economically important traits in salmonid breeding programs (*e.g.*, Ødegård *et al.* 2014; Tsai *et al.* 2015, 2016a; Vallejo *et al.* 2016). An example target trait is resistance to sea lice, since these parasites are the primary constraint to production and result in enormous economic, welfare, and environmental cost (Gharbi *et al.* 2015; Tsai *et al.* 2016a). Genomic prediction of sea lice resistance improves selection accuracy by 27% compared to traditional pedigree-based approaches, highlighting the utility of this technique in aquaculture breeding (Tsai *et al.* 2016a). In parallel, a high quality reference genome assembly has been developed for Atlantic salmon (Lien *et al.* 2016), and the SNP arrays have been integrated with this recent assembly (Lien *et al.* 2016; Tsai *et al.* 2016b). This combination of tools now facilitates the study and use of genotype imputation approaches to improve genomic selection. The potential of genotype imputation in salmon was highlighted in a recent study by Kijas *et al.* (2016), who imputed from low density (0.5–5 K) up to high density (78 K) with high accuracy (0.89–0.97) based on a multi-generation reference population.

The primary goal of the current study was to evaluate the utility of genotype imputation in a population of Atlantic salmon from a commercial breeding program, for which high density genotype information was available on parents and offspring (two generations only). A large proportion of SNP genotypes were masked in the offspring, resulting in “pseudo” low density panels. The correlation between true genotypes and imputed genotypes was then assessed for the masked SNPs under various scenarios. Finally, the imputed SNP data were used in genomic prediction for key economic traits, and prediction accuracy was assessed relative to pedigree-based approaches, and genomic approaches using the full genotype dataset.

## Materials and Methods

### Animals and phenotypes

The genotype and trait data used in the current study were from 624 Atlantic salmon postsmolts, which was a sample from a specific year group subset of a large commercial breeding program (Landcatch Natural Selection Ltd., UK) hatched in the spring of 2008. The samples comprised 59 nuclear families, derived from 30 sires and 59 dams. At ∼1 yr posthatching, juvenile fish were challenged with sea lice (*L. salmonis*) copepods as described in Gharbi *et al.* (2015) and Tsai *et al.* (2016a). Briefly, all fish were challenged in a single tank with a dose of 96 copepod larvae per fish, and monitored until lice had moulted into chalimus I (7 d postchallenge), at which stage fish were measured for body weight (grams), and number of lice attached to the fish (lice were identified by stereo-microscopic inspection, Olympus SZ-40). Therefore, the two phenotypes used in the current study were sea lice counts and body weight, as described in Tsai *et al.* (2016a). Both these traits have been shown in previous studies to be heritable, but with a predominantly polygenic genetic architecture (Ødegård *et al.* 2014; Tsai *et al.* 2015, 2016a; Correa *et al.* 2016). The sea lice count data were transformed to account for a positively skewed distribution, using the approach of Gjerde *et al.* (2011), as described previously for these data (Tsai *et al.* 2016a).

The pedigrees of the fish were identified using PIT-tagging, and an adipose fin clip of each fish was collected and stored in ethanol for genomic DNA extraction. The challenge experiment was performed by the Marine Environmental Research Laboratory (Machrihanish, UK) under approval of the ethics review committee of the University of Stirling (Stirling, UK), and according to Home Office license requirements. All animals were reared in accordance with relevant national and European Union (EU) legislation concerning health and welfare. Landcatch are accredited participants in the Royal Society for the Prevention of Cruelty to Animals (RSPCA) Freedom Foods Standard, the Scottish Salmon Producers Organization Code of Good Practice, and the EU Code-EFABAR Code of Good Practice for Farm Animal Breeding and Reproduction Organizations.

### SNP marker genotyping

All samples were genotyped using the Affymetrix Axiom 132 K Atlantic salmon SNP chip developed by Houston *et al.* (2014), as described in Tsai *et al.* (2015). The quality control measures resulted in the exclusion of SNPs with Mendelian errors, minor allele frequency (MAF) <0.05, and proportion of individuals with missing genotypes >0.03. The MAF of SNPs were calculated using Plink 1.9 (Purcell *et al.* 2007). SNPs with a known and unique chromosome position on the Atlantic salmon reference genome [GenBank accession GCA_000233375.4, (Lien *et al.* 2016)] were retained for analysis. After these filtering steps, 78,362 (78 K) SNPs were retained for the high density SNP panel (hereafter “HD SNP panel”). A subset of these SNPs [25,634 (25 K)] formed part of a second medium density Affymetrix Axiom array described in Tsai *et al.* (2015), and these formed a medium density SNP panel (hereafter “MD SNP panel”). The details of the SNPs in the MD SNP panel and the HD SNP panel are provided in Supplemental Material, File S1 and File S2, respectively. As a result, all parents and offspring samples had genotypes for both SNP panels (the genotype data are provided in File S3), and these formed the basis of the imputation analyses.

### Genotype imputation analyses

#### Definition of high and low density SNP panels:

To test genotype imputation accuracy, a number of test scenarios were established as outlined below and represented in Table 1. While all individuals were genotyped for both the HD SNP panel and the MD SNP panel, some individuals had a set proportion of genotypes masked to mimic the use of lower density SNP panels (hereafter “LD SNP panels”) data for these individuals *in silico*. For the individuals chosen to have LD SNP panel data, two settings determining the content of the LD SNP panel were applied by masking either 90 or 99% of the markers. The remaining SNPs (10 or 1% of all SNPs, respectively) were selected to be evenly spaced throughout the genome, based on physical distance according to the Atlantic salmon reference genome assembly [GenBank accession GCA_000233375.4 (Lien *et al.* 2016)]. Therefore, since the LD SNP panels were created based on both the HD SNP panel (78 K SNPs) and the MD SNP panel (25 K SNPs), the LD SNP panels corresponded to SNP densities of ∼7836 SNPs, 784 SNPs, 2563 SNPs, and 256 SNPs, respectively (Table 1).

**Table 1:** The SNP genotype densities used for the imputation analyses

Original SNP Panel Used to Genotype All Animals	Genotypes Masked to Mimic LD SNP Panels in Offspring (%)	Number of SNPs in LD SNP Panels in Offspring
High density (78 K)	90	7836
	99	784
Medium density (25 K)	90	2563
	99	256

The original SNP panels were either high density (HD) or medium density (MD), which were masked in a (proportion of) the offspring to mimic genotyping with various low density panels.

#### Proportion of offspring genotyping for LD SNP panels:

For all the marker density settings described above, the parents had either HD or MD SNP panel data, and two scenarios were evaluated, where either (i) all offspring had LD SNP panel data, or (ii) 75% of offspring had LD SNP panel data, and the remaining 25% had MD or HD SNP panel data. The latter scenario was applied to measure the impact of including a proportion of offspring with complete genotype information on the phasing and imputation accuracy. The 75% of offspring chosen for LD panel data in scenario (ii) were evenly distributed across all nuclear families in the population.

#### Evaluation of genotype imputation accuracy:

The genotype imputation analyses were performed using the AlphaImpute v1.3.2 software (Hickey *et al.* 2012b) following the standard procedures, using the “HMM” option (Antolín *et al.* 2017), 10 processor cores, and 5 “InternalIterations.” The “CoreAndTailLengths” and “CoreLengths” were set according to the length of corresponding chromosomes. The imputation accuracy was calculated as the correlation (*r*) between the allele dosage of the true genotype and the most likely imputed genotype, averaged across all SNPs and all animals.

### Genomic prediction accuracy using fivefold cross validation

Due to the fact that medium SNP densities (between 5 K and 20 K SNPs) are sufficient for achieving maximum genomic prediction accuracy in the current experimental set up (Tsai *et al.* 2015, 2016a), only the MD SNP panel (25 K SNPs) was evaluated for testing genomic prediction using imputed genotypes. Genomic breeding values were estimated using best linear unbiased prediction using the genomic relationship matrix to model the polygenic relationship between the animals (GBLUP) using ASReml 3.0 (Gilmour *et al.* 2014). The following animal model was employed:

y=Xb+Za+e

where y is a vector of observed phenotypes, b is the vector of fixed effects (sex), a is a vector of additive genetic effects distributed as ∼$\text{N}\left(0,G\sigma_{\text{a}}^{2}\right)$ or $\text{N}\left(0,A\sigma_{\text{a}}^{2}\right)$ where $\sigma_{\text{a}}^{2}$ is the additive (genetic) variance, G and A are the genomic and pedigree relationship matrices, respectively. X and Z are the corresponding incidence matrices for fixed and additive effects, respectively, and **e** is a vector of residuals. The genomic relationship matrix was constructed using the method of VanRaden (2008), and then inverted by applying the standard R function “solve” (R Core Team 2016).

To test the accuracy of genomic and pedigree-based prediction, a cross-validation approach was applied (as described in Tsai *et al.* 2015, 2016a). Briefly, the individuals with imputed genotypes (progeny) were divided into training (80% individuals) and validation (20% individuals) sets. This process was repeated five times, resulting in nonoverlapping validation sets. The lice count and body weight phenotypes were masked in the five validation sets, and then predicted from the genomic breeding values. The prediction accuracy was measured in the validation sets as the correlation between the genomic breeding values, and the trait values divided by the square root of the heritability$\;\left[r\left(y_{1},y_{2}\right)/h\right].$ The fivefold cross-validation analyses were performed for each level of genotype masking and imputation. In all cases, the LD SNP panels were imputed to the MD SNP panel (25 K SNPs), and this imputed genotype data set was used as the input for the GBLUP calculations.

### Data availability

The data used in this study are available as supplementary files. File S1 contains details of the SNPs used for the medium density (25 K) SNP platform. File S2 contains details of the SNPs used for the high density (78 K) SNP platform. File S3 contains the family and phenotype data used in the analysis. File S4 contains the genotype data used in the analysis.

## Results and Discussion

### Accuracy of imputation

#### Comparison of high and medium density SNP panels:

The accuracy of imputation of high density genotypes was assessed as the correlation between the imputed genotypes and the true genotypes in the offspring, where varying proportions of genotypes had been masked. The imputation accuracy ranged from 0.62 to 0.85 for the MD SNP panel (25 K), and from 0.76 to 0.90 for the HD SNP panel (78 K). The higher imputation accuracy based on the HD panel compared to the MD panel may be explained by more accurate resolution of haplotypes, especially for short chromosome segments. Higher imputation accuracy with increased marker density has been shown previously in simulated and experimental populations in livestock (*e.g.* Hayes *et al.* 2012) and crops (Hickey *et al.* 2012a).

#### Comparison with previous studies:

The imputation accuracies achieved in the present study (ranging from 0.62 to 0.90) were generally lower that that achieved in previous studies in farmed animals and crops (*e.g.*, Hickey *et al.* 2012b; Segelke *et al.* 2012; Pausch *et al.* 2013; Moghaddar *et al.* 2015; Uemoto *et al.* 2015; Kijas *et al.* 2016). It is possible that the modest sample size of the current study (*n* = 624) may have been a limiting factor in determining the imputation accuracy. In addition, the lack of genotyped ancestral generations may have impaired the phasing of the parental haplotypes for whole chromosomes. When genotype imputation is employed in livestock populations, multiple generations of ancestral genotyped individuals, and pedigree information are typically available. Likewise, in the study of Kijas *et al.* (2016), multiple generations of genotyped individuals were available for the Tasmanian salmon breeding population. These genotyped multi-generation pedigrees are more amenable to resolving the phase of whole chromosome haplotypes, and therefore result in more accurate genotype imputation.

#### Relationship between MAF and imputation accuracy:

**Figure 1:**
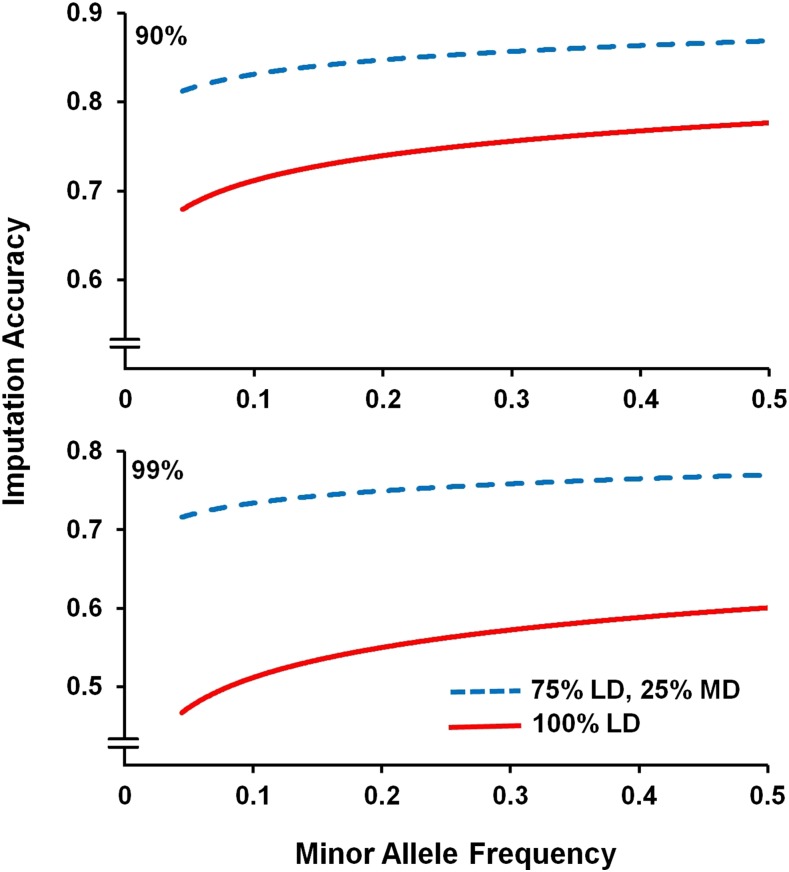
The effect of minor allele frequency on imputation accuracy. The plot shows the imputation accuracy for the MD SNP panel with the two different LD SNP panel densities (90% SNPs masked = 2563 SNPs; 99% SNPs masked = 256 SNPs), plotted against the minor allele frequency of the SNPs using a local regression fit.

The relationship between SNP MAF and imputation accuracy was assessed under four scenarios using the MD SNP panel, varying the density of the LD SNP panel (either 90 or 99% of SNPs masked to mimic LD panels), and the proportion of offspring designated as being genotyped for the LD panels (100 or 75%). Under these scenarios, the correlation between true and imputed genotypes increased with higher MAF (and it should be noted that SNPs with MAF <0.05 had already been filtered out prior to this analysis; Figure 1). This relationship between MAF and imputation accuracy is consistent with previous studies, where accuracy was higher for common variants (*e.g.*, Ma *et al.* 2013; Pausch *et al.* 2013). It is anticipated that the imputation accuracy for rare alleles (and rare haplotypes) will improve with increased sample size, due to the increased frequency of observing these alleles, and with a multi-generation pedigree structure amenable to resolving whole chromosome haplotypes. As expected, including a higher number of SNPs in the LD panel (*i.e.*, 90% masked) resulted in higher imputation accuracy at all MAF (Figure 1 and Table 2).

**Table 2:** Summary of genotype imputation accuracy

SNP Panel	Offspring Genotyping Strategy	Genotypes Masked to Mimic LD SNP Panels in Offspring	
		90%	99%
High density (78 K)	100% LD	0.85	0.76
	75% LD and 25% HD	0.90	0.85
Medium density (25 K)	100% LD	0.76	0.62
	75% LD and 25% MD	0.85	0.75

The correlation between true genotypes and imputed genotypes is presented based on genotype data from the HD SNP platform (78 K) and the MD SNP platform (25 K), with either 90 or 99% of genotypes were masked in the offspring to mimic LD SNP platforms (Table 1). The proportion of offspring genotyped for the LD SNP platforms was either 100 or 75%.

#### Variation in imputation accuracy across animals:

**Figure 2:**
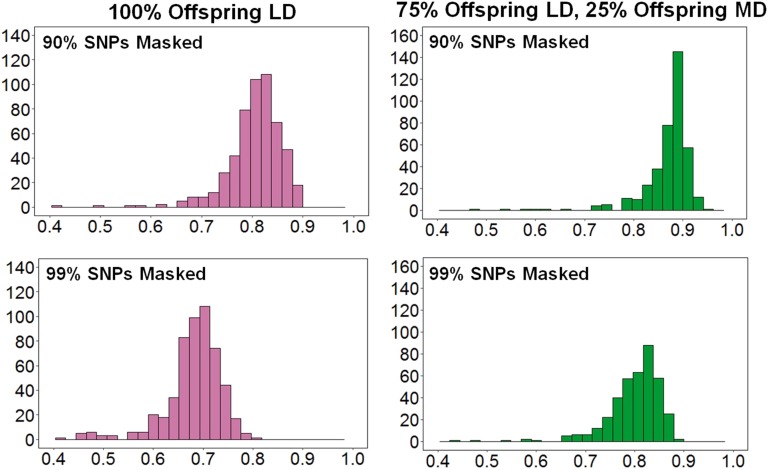
Variation of imputation accuracies across individual animals in MD SNP panel. The histograms show bins of imputation accuracy (*x*-axis), and the number of animals in those bins (*y*-axis) for the two different LD SNP panel densities (90% SNPs masked = 2563 SNPs; 99% SNPs masked = 256 SNPs).

The mean imputation accuracy using the HD SNP panel when all offspring were designated as being genotyped for the LD panels was 0.76, increasing to 0.85 when only 75% of the offspring were genotyped at LD (and 25% genotyped at HD). The equivalent figures for the MD SNP panel were 0.62 and 0.76, respectively. However, there was a large degree of variability of imputation accuracy across individuals, demonstrating a negatively skewed distribution (Figure 2). While the majority of the offspring with imputed genotypes had accuracy values in the range of 0.7–0.9, there was a proportion with much lower accuracy, which reduced the mean accuracy values. This phenomenon has also been reported in studies of imputation in livestock (Hickey *et al.* 2012b; Moghaddar *et al.* 2015), and may arise because certain individual parents have inferior definition of whole chromosome haplotypes to others. Removal of individuals or SNPs with the least accurate imputation values would increase overall average imputation accuracy, but was not performed in the current study.

### Accuracy of genomic prediction using imputed data

**Figure 3:**
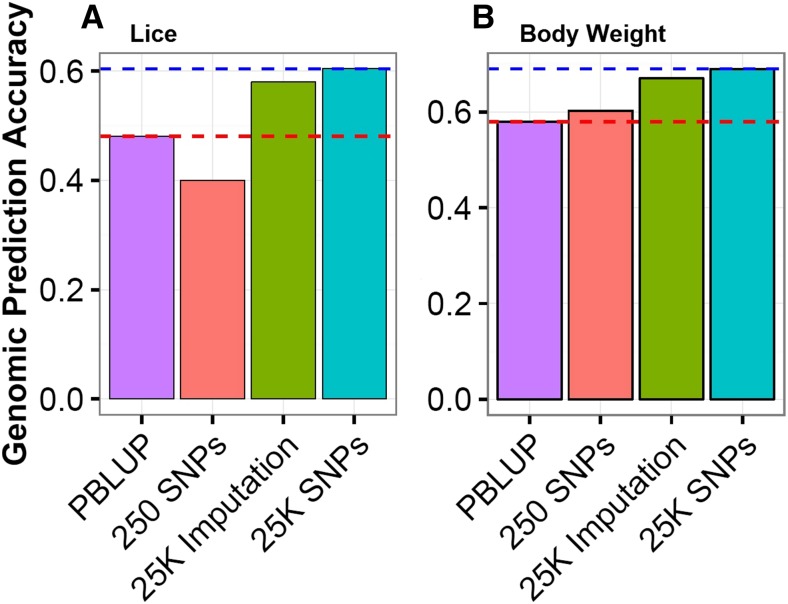
Breeding value prediction accuracies for (A) sea lice resistance and (B) body weight calculated using (i) the pedigree (PBLUP), compared to genomic prediction using (ii) the 256 SNP LD panel only, (iii) the 256 SNP LD panel imputed to 25 K SNPs (with all parents and 25% offspring genotyped at MD SNP panel), and (iv) the true genotypes for the 25 K MD SNP panel. For comparison, the accuracy of breeding value prediction under scenario (iv) is shown by the blue dashed line, and the corresponding accuracy under scenario (i) with the red dashed line.

The second major aim of the current study was to assess the utility of imputed genotype data for genomic prediction in a commercial salmon breeding program. From a practical standpoint, genotyping parents at medium or high density, combined with offspring at lower density with imputation, has potential for major improvement in cost-effectiveness of genomic selection in aquaculture. For the genomic prediction analyses, only imputed data from the MD SNP panel (∼25 K mapped, ordered SNPs) was tested, based on previous studies which suggested that between 5 and 20 K SNPs is adequate for maximum prediction accuracy in a typical salmon breeding set up (Ødegård *et al.* 2014; Tsai *et al.* 2015, 2016a). The imputed genotypes used for genomic prediction were retrieved from the scenario where 75% of offspring were assumed genotyped at LD (and 25% at MD), and the LD SNP panel was created by masking 99% of the MD SNP genotypes (akin to a 256 SNP panel; Table 1). The prediction accuracies using imputed genotypes were marginally lower than tests using true genotypes (0.58 *vs.* 0.60 for lice resistance, 0.67 *vs.* 0.69 for body weight), but substantially higher than pedigree-based method for both phenotypes (0.48 and 0.58 for lice resistance and body weight, respectively) (Figure 3). Taking the pedigree-based breeding value prediction as the baseline, prediction accuracy was improved by nearly 25% when using 25 K true genotypes, and by 21% when using imputed genotypes for the traits of lice resistance (25 K imputation with 75% LD) (Figure 3). This highlights the potential of imputation for cost-effective genomic prediction for the traits studied, although it is important to note that the value of genotype imputation may vary according to the genetic architecture of the trait of interest.

The genomic prediction results are consistent with previous studies of imputation in livestock species, where accuracies using imputed genotypes were slightly lower than those using true genotypes (Berry and Kearney 2011; Segelke *et al.* 2012). Genomic prediction accuracy using just the LD SNP panel (*i.e.*, 256 SNPs) was also compared to prediction accuracy using the LD SNP panel plus imputation. For the trait of sea lice resistance, genomic prediction using 256 SNPs was inferior to pedigree-based prediction (accuracy ∼0.40 *vs.* 0.48), while 256 SNPs with imputation increased the accuracy to 0.58 (Figure 3). For body weight, a similar profile was observed, where pedigree-based prediction accuracy was 0.58, and increased to 0.68 with 256 SNPs and imputation, *vs.* 0.70 with the full 25 K true genotypes. Interestingly, genomic prediction accuracy was generally higher for the trait of body weight compared to sea lice resistance. The heritability of body weight was substantially higher than lice resistance (0.50 *vs.* 0.22; Tsai *et al.* 2016a), which may be expected to result in increased accuracy of genomic prediction (*e.g.*, Sonesson and Meuwissen 2009).

When considering targeted SNP assay genotyping panels (as opposed to direct genotyping by sequencing approaches; discussed briefly below), there is a nonlinear relationship between SNP panel density and cost per sample. This relationship depends on several factors, including the technology, the company, and the number of genotyped samples. However, in general terms, SNP densities of <∼3000 SNPs can be genotyped most cost-effectively using individual targeted assays, for example using KASP technology (LGC Genomics, UK), or targeted genotyping by sequencing (*e.g.*, Affymetrix Eureka technology), while SNP densities >∼3000 SNPs can be genotyped most cost-effectively using SNP arrays. The cost per sample of genotyping for a medium density SNP chip is several fold higher than the cost of genotyping for a 256 SNP panel. Assuming an approximate price for the former of £40 per sample, and an approximate price for the latter of £5 per sample, the total cost of the genotyping for genomic prediction using the imputation described herein is ∼60% lower than genotyping all samples at MD. Furthermore, the efficacy of genotype imputation (and therefore genomic prediction using imputed data) is likely to increase as high density genotype data are collected on additional generations, especially for grandparents of the population where imputation is being applied. The current study used SNP array genotyping data as the basis for imputation, but genotyping by sequencing approaches such as RAD-Seq (Baird *et al.* 2008) have been applied for genomic selection in aquaculture (Dou *et al.* 2016; Vallejo *et al.* 2016; Palaiokostas *et al.* 2016; Robledo *et al.* 2017), and the benefits of a combined high and low density genotyping strategy with imputation may also be relevant to these genotyping techniques.

The focus of this study was to test the possibility of using genotype imputation to improve the cost-efficiency of genomic selection in salmon breeding (by reducing genotyping costs). There are a number of other routes to improving cost-efficiency of genomic selection; for example by preselecting candidates for genotyping based on trait or breeding values (*e.g.*, Lillehammer *et al.* 2013; Ødegård and Meuwissen 2014). Another route to improvement of genomic selection in salmon is to increase overall selection accuracy, particularly where trait records are only available on distant relatives of the selection candidates (Tsai *et al.* 2016a). Successful achievement of accurate “cross-population” genomic prediction reduces the requirement for yearly testing on close relatives (*e.g.*, siblings) of selection candidates. Prediction accuracy in this scenario is likely to benefit large sample sizes for the training populations, high marker density (potentially using low-cost sequencing methods), and/or prioritization of putative functional variants in the SNP panel used for prediction. The latter may be enhanced by initiatives such as the Functional Annotation of All Salmonid Genomes (FAASG; Macqueen *et al.* 2016).

### Conclusion

Genotype imputation approaches were tested in a sample of Atlantic salmon from a commercial breeding program, and the efficacy of using imputed genotype data for genomic prediction was evaluated. Using a two-generation design, with parents genotyped at medium or high density, and offspring genotyped at a lower density, imputation accuracy of up to 0.90 was possible. Genomic prediction accuracy using imputed genotype data were comparable to true genotype data with a ∼250 SNP panel used on 75% of the offspring. However, overall improvement in imputation accuracy may be expected by genotyping additional ancestral generations in the pedigree. Genomic prediction accuracies using imputed genotypes were very close to those using true genotypes, for both growth and sea lice resistance traits. Given that low density genotyping is substantially cheaper than medium or high density, imputation approaches may contribute to the widespread and cost-effective generation of genome-wide SNP data for genomic selection in aquaculture breeding programs.

## Supplementary Material

Supplemental material is available online at www.g3journal.org/lookup/suppl/doi:10.1534/g3.117.040717/-/DC1.
